# Optical Imaging for Monitoring Tumor Oxygenation Response after Initiation of Single-Agent Bevacizumab followed by Cytotoxic Chemotherapy in Breast Cancer Patients

**DOI:** 10.1371/journal.pone.0098715

**Published:** 2014-06-06

**Authors:** Shigeto Ueda, Ichiei Kuji, Takashi Shigekawa, Hideki Takeuchi, Hiroshi Sano, Eiko Hirokawa, Hiroko Shimada, Hiroaki Suzuki, Motoki Oda, Akihiko Osaki, Toshiaki Saeki

**Affiliations:** 1 Department of Breast Oncology, International Medical Center, Saitama Medical University, Hidaka, Saitama, Japan; 2 Department of Nuclear Medicine, International Medical Center, Saitama Medical University, Hidaka, Saitama, Japan; 3 Central Research Laboratory, Hamamatsu Photonics K.K., Hamakita-ku, Hamamatsu, Japan; AMS Biotechnology, United Kingdom

## Abstract

**Purpose:**

Optical imaging techniques for measuring tissue hemoglobin concentration have been recently accepted as a way to assess tumor vascularity and oxygenation. We investigated the correlation between early optical response to single-agent bevacizumab and treatment outcome.

**Methods:**

Seven patients with advanced or metastatic breast cancer were treated with single-agent bevacizumab followed by addition of weekly paclitaxel. Optical imaging of patient's breasts was performed to measure tumor total hemoglobin concentration (tHb) and oxygen saturation (stO_2_) at baseline and on days 1, 3, 6, 8, and 13 after the first infusion of bevacizumab. To assess early metabolic response, 2-deoxy-2-(^18^F)-fluoro-D-glucose (FDG) positron emission tomography/computed tomography (PET/CT), ^18^F-fluoromisonidazole (FMISO)-PET/CT, and magnetic resonance imaging were performed at baseline and after two cycles of the regimen.

**Results:**

Seven patients were grouped as responders (n = 4) and nonresponders (n = 3) on the basis of metabolic response measured by FDG-PET/CT. The responders showed remarkable tumor shrinkage and low accumulations of FMISO tracer relative to those of the nonresponders at the completion of two cycles of chemotherapy. Tumors of both groups showed remarkable attenuation of mean tHb as early as day 1 after therapy initiation. The nonresponders had lower baseline stO_2_ levels compared with adjacent breast tissue stO_2_ levels along with a pattern of steadily low stO_2_ levels during the observation window. On the other hand, the responders appeared to sustain high stO_2_ levels with temporal fluctuation.

**Conclusions:**

Low tumor stO_2_ level after single-agent bevacizumab treatment was characteristic of the nonresponders. Tumor stO_2_ level could be a predictor of an additional benefit of bevacizumab over that provided by paclitaxel.

## Introduction

Bevacizumab, a monoclonal antibody against vascular endothelial growth factor (VEGF) A, has demonstrated clinical efficacy in combination with chemotherapy in patients with HER2 negative breast cancer [1],[2]. To date, although it is believed that a particular subset of patients could greatly benefit from early adoption of bevacizumab in addition to chemotherapy, no specific biomarkers for assessing bevacizumab response have been consistently validated [3].

Diffuse optical spectroscopic imaging (DOSI) is a noninvasive imaging technology using near-infrared light that can measure tissue hemoglobin concentration obtained from spectroscopic oxy-hemoglobin (O_2_Hb) and deoxy-hemoglobin (HHb) data as well as directly visualize vascularity and tissue oxygenation indicated from tHb (O_2_Hb+HHb) and stO_2_ (O_2_Hb/tHb), respectively [4],[5]. DOSI has been currently integrated into several clinical neoadjuvant studies that have explored hemodynamic biomarkers for predicting early treatment response [6],[7],[8].

Zhu et al. reported that remarkable reduction in tumor tHb of primary breast cancer after early treatment cycles of neoadjuvant chemotherapy could predict favorable pathological outcome [7]. In a separate study, Roblyer et al. reported that transient increase in O_2_Hb on day 1 after chemotherapy initiation was characteristic of responders but not nonresponders [8]. These results suggested the clinical importance of tumor oxygenation response to chemotherapy sensitivity. Jain first proposed a therapeutic concept with bevacizumab involving a “normalization window” of tumor vasculature in which more accurate remodeling of the disorganized structure and abnormal functioning of tumor vessels would improve perfusion and enhance tissue oxygenation, which would result in more efficient delivery of cytotoxic drugs [9]. We hypothesized that if vascular normalization occurs after successful vascular remodeling, tumor tHb level should decrease and stO_2_ level should simultaneously improve.

In this clinical study, we used DOSI to monitor tumor mean tHb and stO_2_ levels after the initiation of single-agent bevacizumab followed by cytotoxic chemotherapy in patients with advanced or metastatic breast cancer and determined if early changes in tHb and stO_2_ over a period of single-agent bevacizumab administration could be a predictor of treatment response.

## Materials and Methods

From October 2012 through December 2013, we enrolled patients with locally advanced or metastatic HER2-negative breast cancer (TNM stage III or IV) to receive a combination chemotherapy regimen with paclitaxel and bevacizumab. Patients who have received prior chemotherapy or hormonal therapy before participating in this study were also included. Patient history, including histopathological and radiological imaging results and Ki67 proliferative index, was obtained from medical records. The treatment regimen reported in the study was standard care.

This study was approved by the institutional review board of the International Medical Center, Saitama Medical University, and written informed consent was obtained from each participant prior to inclusion (12-084).

### Chemotherapy regimen

**Figure 1:**
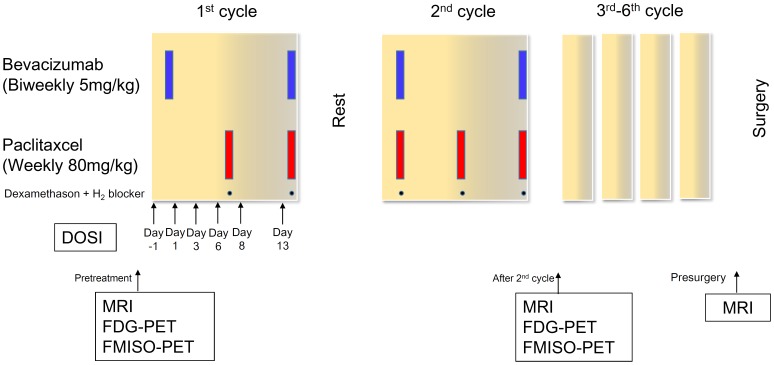
The schedule of treatment and imaging tests. Schematic of combination treatment for patients with advanced or metastatic breast cancer. All patients received bevacizumab (5 mg/kg body weight) intravenously on days 1 and 15 in combination with paclitaxel (80 mg/m^2^ body surface area) on days 1, 8, and 15 and repeated every 4 weeks. Subsequently, the patients underwent 5 additional cycles of bevacizumab and paclitaxel. Both before treatment and after the 2^nd^ cycle of chemotherapy, the patients underwent MRI, FDG-PET/CT, and FMISO-PET/CT. Imaging using diffuse optical spectroscopy was also performed on day 1 before the first infusion of bevacizumab and on days 1, 3, 6, 8, and 13 after the infusion.

All patients received bevacizumab (5 mg/kg body weight) intravenously on days 1 and 15 in combination with paclitaxel (80 mg/m^2^ body surface area) on days 1, 8, and 15, repeated every 4 weeks (Figure 1) [10]. Paclitaxel infusion was omitted on the first day of the first cycle. Dexamethasone (6.6 mg) and an H_2_ antagonist were used for supportive treatment during the course of chemotherapy; however, use of these drugs in the first infusion of bevazicumab was omitted. Breast surgery was performed for patients deemed resectable after 5–6 weeks of completion of the initial chemotherapy. Treatment continued for six cycles unless there was disease progression, unacceptable toxicity, or withdrawal of consent. If study treatment was discontinued, further local and/or systemic treatment was permitted at the investigator's discretion.

### TRS breast imaging system

To extract quantitative hemoglobin concentrations from breast tissue, we developed a system that used a time-correlated single-photon counting (TCSPC) method for measuring temporal response profiles of tissue against optical pulse inputs and enabled quantitative analysis of light absorption and scattering in tissue according to the photon diffusion theory [4],[11]. This approach could quantify O_2_Hb and HHb tissue levels. Details of the TRS breast imaging system have been previously published [12].

An ultrasound-assisted optical probe was used to visualize the largest tumor lesions, which were located in the center of a 10-mm square grid map that was constructed for the lesion and surrounding normal tissue. The grid map of a tumor-bearing breast basically comprised 7×7 points with a 10-mm interval between two points in the x–y dimension. For spline interpolation, custom software (DataBreastViewer, version 109; SincereTechnology Corp., Kanagawa, Japan) was used to perform 2D image processing and analysis.

### Hemodynamic biomarkers

The distribution of tHb levels of a tumor-bearing breast shows the functional vascular tumor volume, which is contrasted by that of the surrounding normal tissue. The distribution of stO_2_ levels maps the magnitude of tissue oxygenation of breast tissue. A lesional region of interest (ROI) 2 cm in radius from the center of the tumor was constructed, and the mean levels of tHb and stO_2_ were calculated. To demonstrate the capacity of DOSI to reveal the tumor response of bevacizumab, we monitored changes in the mean levels of tHb and stO_2_ of a tumor-bearing breast and a normal contralateral breast at baseline (day -1) and on days 1, 3, 6, 8, and 13 after the first infusion of bevacizumab.

### Serial examination of positron emission tomography (PET)/computed tomography (CT) using 2-deoxy-2-(^18^F)-fluoro-D-glucose (FDG) and ^18^F-fluoromisonidazole (FMISO)

Biograph 6 (Siemens, Medical Systems, Inc.:Suite, Washinton, D.C., United States) was used to perform PET/CT. Details of the FDG PET/CT procedure have been described previously [13] [14]. All patients were required to fast for at least 6 h to confirm normal glucose blood levels. One hour after the administration of FDG tracer (3.7 MBq/kg), the patients were positioned prone on the whole-body PET/CT scanner couch. CT was initially performed followed by a PET emission scan that covered the identical transverse field of view. The Biograph allows simultaneous collection of 16 slices over a span of 15.8 mm with a slice thickness of 2.5 mm and a transaxial resolution of 6.3 mm. The acquisition time was 2 min per table positron. PET scans were processed, reconstructed with an ordered subset expectation maximization, and measured attenuation correction. Ordered subset expectation maximization image reconstruction was used for all data. Acquisition of PET data was operated in three-dimensional and high resolution mode.

According to protocol, FMISO-PET/CT scan was basically performed 1 day after the FDG-PET/CT scan. All patients were intravenously injected with 7.4 MBq/kg of FMISO. At 2 h after injection, FMISO-PET/CT was performed immediately after the CT scan. ROIs with 1.0-cm maximum diameters were drawn on the areas of abnormal FDG or FMISO accumulation corresponding to the baseline tumor lesions. In a series of PET/CT scans, care was taken to draw the ROI in the same lesion as shown on the baseline lesion. For deciding each ROI, CT combined with PET provided anatomical landmarks for detecting the lesion, and the maximal standardized uptake value (SUVmax) was recorded from the target. The PET/CT images were analyzed by at least two radiologists in a blinded manner. SUV was calculated according to the following formula:

SUV = activity concentration in ROI (MBq/ml)/injection dose (MBq/kg body weight). The serial FDG and FMISO PET/CT scans were scheduled before treatment initiation and after two cycles of chemotherapy. PET/CT scans were performed at least 2 weeks after performing a diagnostic core biopsy and after infusion of drug.

### Serial examination using breast magnetic resonance imaging (MRI)

Details of the breast MRI have been described previously [15]. The device used was a 1.5-T instrument (Avanto; Siemens, Erlangen, Germany) that used a body coil for transmission and a two-channel breast array coil for reception. For measuring maximum diameters of lesions, post-contrast coronal, axial, and sagittal images obtained by contrast-enhanced dynamic imaging were used. The percentage changes in tumor maximal size at baseline and after two cycles for lesions were assessed radiographically.

### Study end points

**Figure 2:**
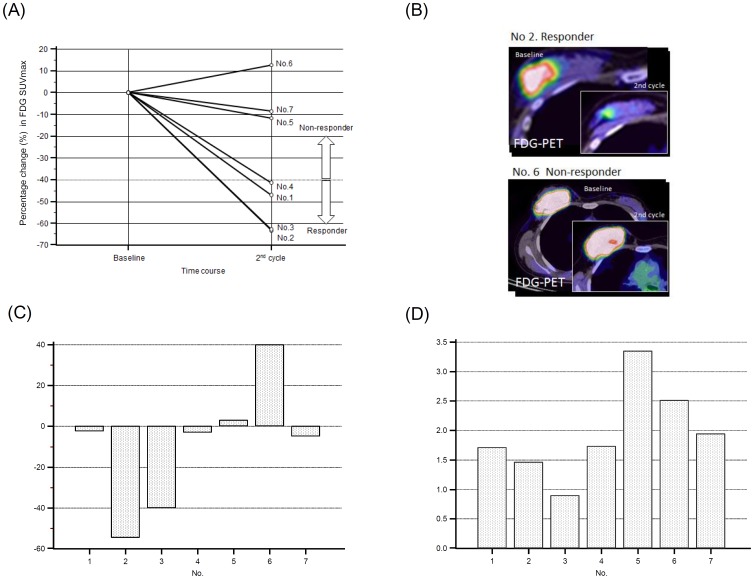
Results of tumor shrinkage and hypoxia after 2^nd^ cycle of chemotherapy. A. Percentage change in 18F-fluorodeoxyglucose uptake expressed as standardized uptake value (%SUV) from baseline after 2^nd^ cycle of chemotherapy regimen. Patients with %SUV >-40% were considered as responders and others were as nonresponders. B. Representative examples of transversal fused PET/CT scans at baseline and after 2^nd^ cycle of chemotherapy with responders (No. 2) and nonresponders (No. 6) are shown. C. Percentage change in maximal size as measured by breast MRI at baseline and after 2^nd^ cycle of chemotherapy. Patient Nos. 1, 2, 3, and 4 were responders and Nos. 5, 6 and 7 were nonresponders. There were no significant difference between responders (mean −24.9±26.4 SD) and nonresponders (mean 12.7±23.9 SD, p = 0.1). D. Lesion maximal SUV (SUVmax) as measured by FMISO-PET/CT at the completion of the 2^nd^ cycle of chemotherapy. The intensity of SUVmax indicates hypoxic activity of the tumor. Nonresponders (mean 2.6±0.7 SD) had significantly higher FMISO-SUVmax than responders (mean 1.4±0.4 SD, p = 0.03)

The schedule of imaging studies using DOSI, MRI, FDG-PET/CT, and FMISO-PET/CT is shown in Figure 1. Early tumor metabolic response assessed by serial FDG-PET/CT has been widely accepted as predictive of clinicopathological outcome for advanced breast cancer in a neoadjuvant setting [16],[17]. An optimal cutoff value between 40% and 65% of the baseline SUV has been suggested for potential early identification of nonresponders. In this study, we employed a reduction rate of 40% as a cutoff value for FDG-SUV to separate responders from nonresponders (Figure 2A).

### Criteria for morphological response

Patients underwent MRI tests to compare size reduction between baseline and the completion of treatment according to the RECIST guideline [18]. The protocol stipulated that the initial treatment should be discontinued in patients who showed progressive disease (PD) (20% or more increase in size) or appearance of new metastatic lesions and that such patients should be excluded from further participation in the study.

### Assessment of pathological response

Pathological tumor response was determined on the basis of the General Rules of Criteria and Pathological Recording of Breast Cancer 2007 by at least two pathologists [19]. Surgical specimens were cut in 0.5-cm-thick slices and evaluated for the presence of microscopic tumor cells in the invasive area as well as areas with marked fibrosis or scarring, and the presence of ductal component and lymph nodal metastasis was not evaluated. No residual invasive cancer cells in all areas of the surgical specimens was defined as pathological complete response or pCR (grade 3). The disappearance or marked degeneration of two-thirds or more of the tumor cells was defined as substantially effective (grade 2). The disappearance or marked degeneration of one-third to less than two-thirds was defined as moderately effective (grade 1b). The disappearance or marked degeneration of less than one-third of the tumor cells or mild tumor cell degeneration, regardless of the percentage, was defined as mildly effective (grade 1a). Almost no change in cancer cells after treatment was defined as not effective (grade 0).

### Statistics

MedCalc software (Mariakerke, Belgium) was used to perform statistical evaluations. Continuous variables were presented as means, median, and standard deviations (SD). A paired Student's *t*-test was used to analyze changes in variables. Differences at a p value of less than 5% were considered to be statistically significant.

## Results

### Baseline characteristics

We enrolled seven women in this study. All patients received at least two cycles of the regimen. One patient (No. 3) declined further treatment after the completion of two cycles and was therefore withdrawn from the study. Another patient (No. 6) discontinued the study treatment because of a paclitaxel-induced infusion reaction and then received further chemotherapy using nab-particle paclitaxel on a triweekly basis. All patients underwent DOSI, MRI, FDG-PET/CT, and FMISO-PET/CT imaging tests on treatment.

The baseline characteristics of the seven patients are shown in Table 1. The median age of the patients was 52.6 years (range, 36–63 years), and the median tumor size was 51.7 mm (range, 30–67 mm) assessed by breast MRI. Based on breast MRI and FDG-PET/CT examination, five patients were determined to have TNM stage III and two patient had stage IV with lung and multiple bone metastases. One patient (No. 1) had previously received hormonal therapy, and the other six patients had not previously received systemic therapy. Five patients had histologically invasive ductal carcinoma (IDC) with luminal subtype, and two patients had IDC with triple-negative subtype.

**Table 1:** Patient demographics.

No.	Age (year)	Rt/Lt	Histology	Size (mm)	TNM stage	ER (%)	PgR (%)	HER2	Ki67 (%)
1	58	Lt	IDC	43	T4N1M1	50	0	0	30
2	51	Rt	IDC	44	T4N1M0	90	50	1	35
3	45	Lt	IDC	30	T4N1M0	90	10	1	20
4	56	rt	IDC	67	T3N1M0	0	0	1	15
5	61	Rt	IDC	67	T3N1M0	90	90	1	20
6	36	Rt	IDC	50	T4N3M0	50	50	1	70
7	63	Lt	IDC	61	T4N1M1	0	0	0	65

Lt, left; Rt, right; IDC, invasive ductal carcinoma; ER, estrogen receptor; PgR, progesteron receptor, HER2; c-erb-B 2.

### Assessment of therapeutic response

Employing a reduction rate of 40% as a cutoff value for FDG-PET/CT metabolic response, tumors with reductions ≥40% were defined as responders (n = 4), and tumors with <40% reductions were defined as nonresponders (n = 3) (Figure 2A and B). Figure 2C showed that the responders achieved remarkable tumor shrinkage (mean, −24.9%±26.4 SD) and Figure 2D showed low levels of FMISO-SUV (mean, 1.4, ±0.4 SD) at the completion of the 2^nd^ cycle of chemotherapy. On the other hand, the nonresponders showed no evidence of tumor shrinkage (mean, 12.7%±23.9 SD) but still sustained high levels of FMISO-SUV (mean, 2.6±0.7 SD) at the same time point. There was significant difference of FMISO-SUV level between responders and nonresponders (p = 0.03).

All nonresponding patients included PD on the basis of RECIST criteria and/or histopathologically no response (grade 0) following surgery. On the other hands, all responding patients included partial response (PR) and/or histopathologically marked or moderate response (grade 2 or grade 1b) (Table 2).

**Table 2:** Therapeutic outcome of patients after administration of the initial chemotherapy.

No.	Completed cycles of initial chemotherapy	% change in tumor size after initial chemotherapy	RECIST criteria	Intervention after initial chemotherapy	Post-surgical pathological assessment	Baseline FDG-SUV_max_	% change in SUV_max_ at 2^nd^ cycle	Metabolic response at 2^nd^ cycle	Comments
1	6	−25.6	PR	Surgery	Grade 2	9.8	−47.1	Response	The patient received endocrine therapy and showed no progression of disease for 6 months after surgery
2	6	−54.5	PR	Surgery	Grade 2	8.5	−63.3	Response	The patient received adjuvant endocrine therapy and no evidence of relapse was found by routine imaging for 8 months after surgery
3	2	−40	PR	-	-	10.3	−63	Response	The patient declined further treatment after the completion of 2 cycles and was therefore withdrawn from the study
4	6	−25.4	PR	Surgery	Grade 1b	4.4	−41.4	Response	The patient received adjuvant endocrine therapy and no evidence of relapse was found by routine imaging for 7 months after surgery
5	5	−13.4	SD	Surgery	Grade 0	6.8	−11.8	Nonresponse	The patient received adjuvant endocrine therapy and locoregional radiotherapy and no evidence of relapse was found by routine imaging for 7 months after surgery
6	2	40	PD	Surgery	Grade 0	23.2	12.7	Nonresponse	The patient underwent adjuvant chemotherapy and locoregional radiotherapy, but relapse in the lung and bones was detected by CT scans 6 months after surgery.
7	2	−4.9	PD	2^nd^ line chemotherapy	-	17.8	−8.5	Nonresponse	Disease progression of primary and metastatic lesions of the lung and bones was detected by FDG-PET/CT scan after administration of 2 cycles of the initial chemotherapy

PR,partial response; SD,stable disease; PD,progressive disease; Grade 2, disappearance or marked degeneration of two-thirds or more of tumor cells; Grade 1b, disappearance or marked degeneration of one-third to less than two-thirds of tumor cells; Grade 0, no change in tumor cells.

### Assessment of hemodynamic biomarkers

**Figure 3:**
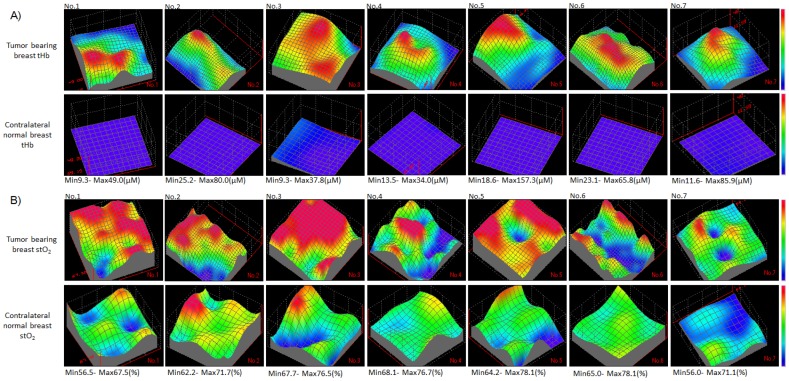
Baseline maps of breast tHb and stO_2_. Three dimensional reconstruction mapping of baseline tHb (A) and stO_2_ (B) of tumor-bearing breast and contralateral normal breast. Tumor-bearing breast map shows a 6×6-cm measurement area that included the tumor located at the center and surrounding normal tissues at the margins. Contralateral normal breast map includes a 4×4-cm measurement area corresponding a mirror image location.

Figure 3A and B showed three dimensional reconstruction mapping of tHb and stO_2_ levels of tumor-bearing breast and contralateral normal breast. All patients had a hotspot corresponding to tumor lesion and significantly higher tHb of tumor-bearing breast (mean, 60.5 µM±38.5 SD) compared with that of contralateral normal breast (mean, 15.2 µM±5.1 SD, p = 0.009). Responding tumors (mean, 37.2%±9.5 SD) had significantly lower level of tHb compared to nonresponding tumors (mean, 91.6%±42.1 SD, p = 0.04). In contrast, for stO_2_ maps, there was no difference between tumor (mean, 67.3%±9.3 SD) and normal tissue (mean, 67.3%±5.1 SD, p = 0.9). Responding tumors appeared an elevation of stO_2_ level corresponding to tumor lesion and surrounding normal tissue, while nonresponding tumors showed a dip of stO_2_ level compared to surrounding normal tissue.

**Figure 4:**
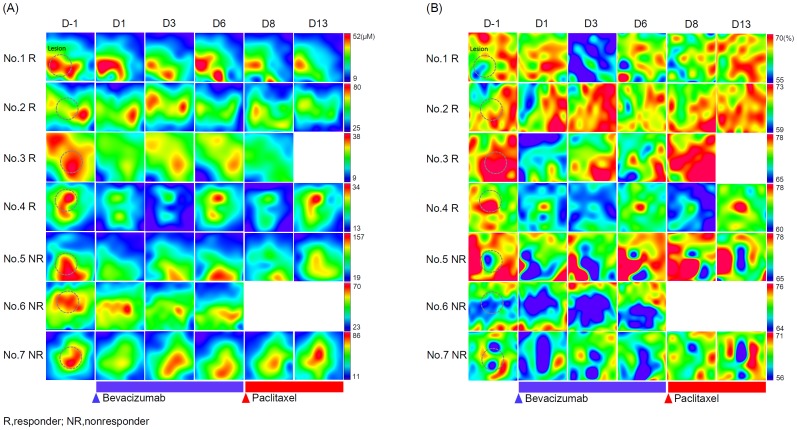
Serial maps of tumor-bearing breast tHb and stO_2_ on treatment. Serial maps of tumor-bearing breast tHb (A) and stO_2_ (B) during bevacizumab treatment at baseline (day −1) and on days 1, 3, 6, 8, and 13 after the initiation of bevacizumab. The measurement points of stO_2_ were exactly identical to those of tHb during treatment.

Figures 4 present serial maps of the distribution of tHb (A) and stO_2_ (B) levels in tumor-bearing breasts of all the patients over the observed time window during treatment.

**Figure 5:**
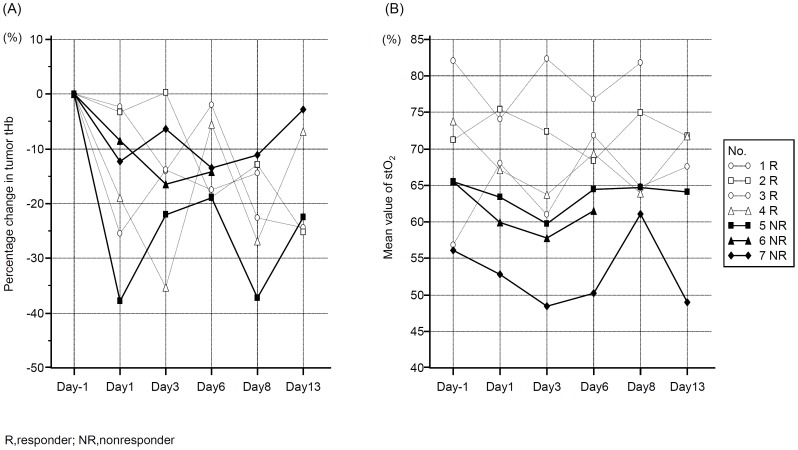
Results of serial monitoring of tumor tHb and stO_2_ during bevacizumab treatment. A. Percentage change in tumor tHb on days 1, 3, 6, 8, and 13 after the initiation of bevacizumab relative to the baseline level. There were no significant difference of the value between responders and non-responders during the time points. B. The mean value of stO_2_ at baseline (Day −1) and on days 1, 3, 6, 8, and 13 after the initiation of bevacizumab. The value of nonresponders was significantly lower than that of responders on day 1, day 3, and day 6 (p<0.05).

Figure 5A shows the change in the mean tumor tHb levels from baseline during treatment. The mean tumor tHb level at baseline (mean, 60.5 µM±38.5 SD) was compared with those at day 1 (mean, 49.6 µM±26.3 SD, p = 0.5), day 3 (mean, 47.5 µM±32.3 SD, p = 0.5), day 6 (mean, 54.6 µM±41.9 SD, p = 0.7), day 8 (mean, 50.5 µM±30.9 SD, p = 0.6), and day 13 (mean, 58.8 µM±40.7 SD, p = 0.9). There were no significant difference of tumor tHb level between responders and nonresponders on each time point.

Figure 5B shows the observed mean tumor stO_2_ percentage at baseline and during the treatment. The mean stO_2_ percentage at baseline did not differ between responders (mean, 70.9%±10.5 SD) and nonresponders (mean, 62.4%±5.4 SD, p = 0.1), but the mean stO_2_ percentage was significantly higher in responding tumors at day 1 (mean, 71.1%±4.2 SD), day 3 (mean, 69.9%±9.6 SD), day 6 (mean, 71.6%±3.7SD) compared to nonresponding tumors at day 1 (mean, 58.7%±5.4 SD, p = 0.02), day 3 (mean, 55.3%±6.0 SD, p = 0.04), day 6 (mean, 58.7%±7.5 SD, p = 0.03). There were no significant difference at day 8 and 13 between two groups.

## Discussion

In this study, the breast cancer patients showed considerable variation in the early responses to single-agent bevacizumab. Our initial experience illustrates at least two patterns of tumor hemodynamics. When the patients were grouped into responders and nonresponders on the basis of the serial FDG-PET/CT results, both groups showed remarkably elevated tHb in lesions relative to the adjacent breast tissue tHb levels. Figure 5A shows that a transient decrease from the baseline tumor tHb level occurred in the responders and nonresponders during the first 1 weeks. This finding indicates that a decrease in tumor vascularity could not be a biomarker of substantially therapeutic response to bevacizumab but may be an indicator of other pharmacological effects of bevacizumab; for example, blockage of VEGF, which induces vasoconstriction [20]. Van der Veldt et al. reported that patients who received single-agent bevacizumab had a significant decrease in plasma levels of circulating VEGF at 3 h after bevacizumab administration and the majority of patients had a substantial recovery in VEGF level after 4 days [21]. This trend of circulating VEGF level is consistent with our results of early tHb response to bevacizumab.

Figure 3B shows that the baseline stO_2_ values of nonresponding tumors (Nos. 5, 6 and 7) were lower than those of the surrounding normal breast tissues, and sequential maps of figure 4B showed that relatively low levels of tumor stO_2_ were sustained over the observation time window. In fact, these two patients had unfavorable pathological outcomes based on surgical findings and the other patient had progression of both primary and distant metastatic lesions after treatment. This result may indicate that severe chronic hypoxia was present at baseline with insufficient vascular remodeling and failure to normalize even after infusion of bevacizumab. On the other hand, the observed mean stO_2_ levels of figure 5B at different time points in responding tumors fluctuated depending on the patient, but they apparently trended higher than those of the adjacent normal tissues during the observation time window. For example, the No. 2 patient had a responding tumor that showed a gradual increase in stO_2_ on days 1 and 3 despite attenuation of tHb. This result may explain the effect of bevacizumab, which contributes to normalization of the tumor vasculature and enhances oxygenation. In fact, the No. 2 patient had a better pathological outcome with minimally invasive components at surgery. In the other patients defined as responders, the tumor stO_2_ levels also varied greatly in response to bevacizumab but remained high during the observation time window. However, these tumors substantially improved stO_2_ after paclitaxel and then achieved favorable pathological results. Thus, the concomitant change in tumor stO_2_ may indicate how efficiently tumor oxygenation has recovered following vascular remodeling due to VEGF blockage.

In a retrospective study that examined 41 breast cancer patients who underwent neoadjuvant chemotherapy, Ueda et al. reported that elevated baseline levels of tumor stO_2_ significantly correlated with pCR [22]. In addition, the investigators claimed that tumor reoxygenation exhibited by elevation of tumor O_2_Hb as early as day 1 after the initiation of cytotoxic chemotherapy, which is called O_2_Hb flare, was adequate to discriminate nonresponding tumors from both partial and complete responders [8]. These findings were consistent with the current result that showed the significance of tumor oxygenation to improve chemotherapy response.

In essence, vascular normalization is considered to occur only in regions of the tumor where the abnormal and immature vasculature of the tumor microenvironment has been corrected by proper dosing and timing of bevacizumab, which would result in sufficient oxygen delivery [23]. In other words, our experience suggests that if vascular remodeling fails, bevacizumab-induced vessel regression may cause more severe hypoxia in the tumor microenvironment. However, a limitation of this preliminary study is that the number of patients was too limited to confirm this speculation. A larger number of patients is needed to verify our findings and provide sufficient evidence to support our speculation concerning the possible effect of bevacizumab in patients with failure of vascular remodeling.

In conclusion, this noninvasive optical imaging technique for visualizing vascularity and oxygenation could be useful for tracking the vascular normalization window. Although our findings are not definitive, the initial results suggest that a further study to identify a particular subset of patients who would benefit from bevacizumab may be beneficial.
